# Robust Automatic Focus Algorithm for Low Contrast Images Using a New Contrast Measure

**DOI:** 10.3390/s110908281

**Published:** 2011-08-25

**Authors:** Xin Xu, Yinglin Wang, Jinshan Tang, Xiaolong Zhang, Xiaoming Liu

**Affiliations:** 1 Department of Computer Science and Engineering, Shanghai Jiao Tong University, NO. 800 Dongchuan Road, Shanghai 200240, China; E-Mails: xuxin0336@gmail.com (X.X.); wang-yl@cs.sjtu.edu.cn (Y.W.); 2 School of Computer Science and Technology, Wuhan University of Science and Technology, NO. 947 Heping Road, Wuhan 430081, Hubei, China; E-Mail: xiaolong.zhang@wust.edu.cn (X.Z.); liuxiaoming@wust.edu.cn (X.L.)

**Keywords:** auto-focus, low contrast, contrast measure, noise reduction

## Abstract

Low contrast images, suffering from a lack of sharpness, are easily influenced by noise. As a result, many local false peaks may be generated in contrast measurements, making it difficult for the camera’s passive auto-focus system to perform its function of locating the focused peak. In this paper, a new passive auto-focus algorithm is proposed to address this problem. First, a noise reduction preprocessing is introduced to make our algorithm robust to both additive noise and multiplicative noise. Then, a new contrast measure is presented to bring in local false peaks, ensuring the presence of a well defined focused peak. In order to gauge the performance of our algorithm, a modified peak search algorithm is used in the experiments. The experimental results from an actual digital camera validate the effectiveness of our proposed algorithm.

## 1. Introduction

With the rapid development of digital image processing techniques, the design of a consumer-level digital camera is inclined to introduce user-friendly cameras which aim to provide easy way to obtain high quality imaging with minimal user intervention in tasks such as auto-focus (AF). The basic idea of AF is to replace the tedious process of manual focusing with automatic adjustment of the lens of the camera to the right position, ensuring the image is well positioned at the focal plane [1]. AF is a key factor affecting the sharpness of a final captured image. It is based on the fact that an object in the image appears the sharpest when it is in focus. Otherwise, image will be blurry in an out of focus position [2]. Thus, the most high quality imaging is obtained in large part by how the lens is adjusted by camera’s AF system to bring an image into focus where sharpness is maximized [3].

Many AF systems were developed in the past, which can be classified into two types of systems: active AF and passive AF [4]. Active AF can be achieved through the use of external sensors or measurement tools. The advantage of active AF lies in its ability to focus under different lighting conditions, particular in a low-level luminance environment. However, due to the high infrared or ultrasound reflectivity of external sensor or measurement tool, they may encounter difficulty when focusing through window or glass [5]. In addition, it is generally costly to calculate the distance between the lens and the object of interest. On the contrary, the calculation of in-focus position in passive AF relies on the image information only, and we do not need to consider the reflectivity in passive AF, because no external sensor is used. As a result, most consumer-level digital cameras deploy passive AF.

**Figure 1.**
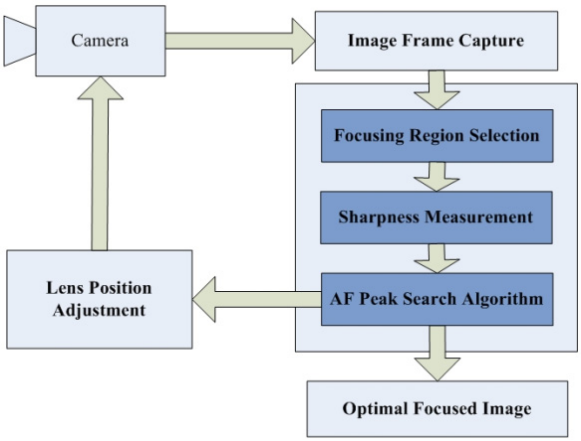
Traditional passive AF system.

A passive AF system generally includes three components: focusing region selection, sharpness measurement, and peak search [4]. As illustrated in Figure 1, a passive AF system normally operates in an iterative manner. First, an image is captured by the camera at the current lens position or focal length. Next, the focusing region selection procedure determines which part of the captured image is used for sharpness computation. Then, a sharpness measurement is applied to that focusing region to calculate a fitness value. Finally, a peak search step is performed to obtain the maximum sharpness or best lens position from the fitness value candidates.

Unfortunately, most passive AF systems do not always work well for low contrast images obtained under dim lighting conditions. Passive AF extracts a measure of sharpness from the image itself, and treats a defocused image as the image generated by convolving a focused image with a low-pass filter whose bandwidth is proportional to the degree of focus [6]. The less an image is focused, the more details it loses. Thus, passive AF usually is based on the high frequency content of the image computed from focus windows, assigning more weight to the high frequency content when evaluating the degree of focus [7]. However, under low light conditions, there is little content in the high frequency range, making it difficult to extract adequate sharpness information. The improvement of low-light AF performance is important for capturing high-quality images to meet consumers’ demand in undesired lighting conditions. The goal of our work described in this paper is to overcome this weakness for most passive AF systems, and propose a robust algorithm which is capable of handling low contrast images in noisy environments.

## 2. Related Works

**Figure 2.**
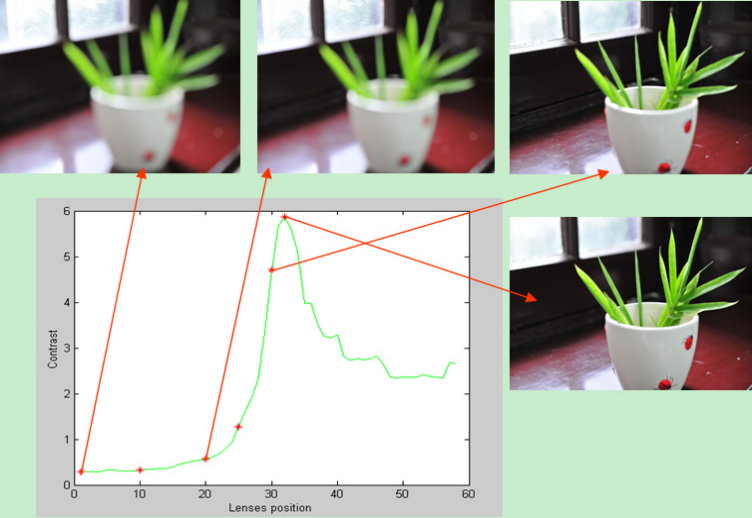
Image contrasts at different lens position.

Sharpness function is a quantitative description of the image sharpness in mathematics, and it is a numerical measure that provides a value indicating the degree of focus for an out-of-focus image [5,8]. Because the sharpness is related to the contrast of an image and thus we can use contrast measure as the sharpness function. As illustrated in Figure 2, the change of contrast accompanies the change of lens position. The contrast reaches a maximum for the best focused image and decreases as the image blurs.

Many contrast measures have been used for passive AF. In [4], comparison of different AF algorithms is made to explore their relative merits through examining them in a variety of scenes. Results indicate that 2D spatial measurement methods such as Tenengrad [9,10], Prewitt Edge detection [11], and Laplacian [6] yield best performance in terms of accuracy and unimodality. However, they are very sensitive to noise, and not robust to different scene conditions such as low light conditions.

On the contrary, variance based methods are fast and robust. The basic idea is to calculate the variance of image intensity. The image is best focused when the variance reaches a maximum [6]. A typical method in discrete cosine transform (DCT) domain is to compute the AC coefficients of images, which can also be used to represent information about variance function of the luminance [12]. However, low light conditions pose a major problem for traditional variance based methods due to its poor discrimination power. In low light conditions, contrast measures become fluctuant, and non-uniformity in image intensity seems to be not distinct when the focus changes. As a result, it is quite difficult to locate the peak.

Recently, several modified variance based methods have been presented for low contrast images. Shen *et al*. [13] scaled the AC coefficients by the overall intensity of image. They calculated both AC coefficients and DC coefficients of image. Then the ratio between AC coefficients and DC coefficients was defined as a contrast measure. The purpose of this method is to compensate and give rise to a homologous region. As a result, this method can work well both for ordinary images as well as low contrast images. Lee *et al*. [14] further improved Shen’s method, which can avoid the iterative moving of step motor. The nonlinear regression routine is used to quickly predict the location of the maximum focus value through a Gaussian profile fitting. Afterwards, Kristan *et al*. [15] proposed a focus measure based on the Bayes spectral entropy of the image spectrum. DCT was used to map between the spatial and spectral domains. The image was divided into non-overlapping sub-images of 8 × 8 pixels, where sharpness values are calculated separately. The mean of the sharpness values is taken as a measure of overall focus. However, this measure needs to establish some predefined threshold, which varies with the content of an image. Lee *et al*. [16] used the ratio of the AC and DC components in DCT as the measure of focus. The lowest five AC components and a DC component coefficient were selected, which contained the most energy (information) of an image as well as the detailed edge and base edge information.

However, due to the neglect of existing correlations among adjacent blocks pixels in DCT computation, it may incur an undesired effect called “blocking artifact” [17]. In addition, the DCT-based methods require much computation time as a lot of processing is involved in conversion to and from the DCT coefficients. Aiming to address this problem, Lin [18] proposed to use regional monotones of focus value variation to attenuate the influence of noise generated false peaks. The inconsistency in the motion direction of lens caused by image disturbances can be checked and corrected using these monotonous properties. However, low illumination could induce a problem of low signal-to-noise (S/N) ratio for focus value measure, which makes it difficult to obtain the monotonous properties because of the noise influence. Afterwards, Gamadia *et al*. [3] processed low contrast images in a different way. They attempted to reduce the effect of the additive white Gaussian noise through introducing image enhancement preprocessing steps. This method is computationally simple, and can improve low light AF performance. However, the discrimination power of their proposed contrast measure is not robust. For a raw image corrupted by another kind of noise except for the additive white Gaussian noise, multiplicative noise for example, their method may fail to work well.

Besides enhancing image contrast, other methods for enabling passive AF in low light conditions include increasing the light intensity via external assist or flash lights [19], increasing the exposure time [20], and increasing the size of the focus window. Unfortunately, these methods all fail to be implemented in consumer-level digital cameras due to different limitations. Although the use of light emitting diodes (LEDs) as an AF assist in digital cameras is a standard feature, their use is not always allowed in some locations such as cinemas and museums [21]. Moreover, the use of external lights may cause a distraction from the moment of a candid shot and result in the degradation of image quality [3]. The increasing of exposure time contributes to the increasing of sensor integration time, and thus enhances the signal to noise ratios (SNR). However, longer sensor integration time may cause the effect of lowering the capture frame rate, and result in undesired motion blur [20]. The increasing of focus window size accompanied the increasing of computation time in contrast measure computation. This may lead to an increase in AF lag time [7].

In this paper, we propose a new passive AF algorithm which is capable of handling low contrast images in noisy environments. A denoising preprocessing is first introduced to reduce the effect of both additive noise and multiplicative noise. Aiming to reduce the difficulty of peak search, a new variance based contrast measure is presented which can further enhance the low contrast image. This contrast measure can carry more information about the image discontinuities, and thus has more discrimination power. Finally, a modified hill climbing algorithm is used to find the focused lens position. Our method was tested on a number of low contrast image sequences captured by an actual digital camera, and result indicates that this algorithm provides an effective means of improving AF performance for low contrast image in noisy conditions. The remainder of this paper is organized as follows. Section 3 proposes a new passive AF algorithm and provides a theoretical analysis of its effectiveness. Experimental results obtained from several image sequences are presented in Section 4. The conclusions are given in Section 5.

## 3. Proposed Passive AF Algorithm

Like the traditional passive AF system illustrated in Figure 1, our proposed AF algorithm also includes three components: focusing region selection, contrast measurement, and peak search. Differently, before proceeding to these components, a noise reduction preprocessing using the modified bilateral filter is introduced to make our algorithm robust to different types of noise. In our study, the region of interest is not restricted to a specific area of the image. Thus we select the entire scene of the image as the focusing region. Then, in order to improve the performance of our AF algorithm under low light conditions, we propose a new contrast measurement which can make the curve peak be easily distinguished from local false peaks generated by noise. Finally, the modified peak search algorithm is presented to gauge the improvement in performance.

### 3.1. Noise Reduction Preprocessing

As stated previously, most of current noise reduction preprocessing for low contrast image is performed under the assumption of an additive noise model, for example, additive white Gaussian noise [3,13]. In our previous work [22], additive noise was removed by a Wiener filter, which is the optimal linear filter for removing additive noise in the mean square error sense [23,24]. However, it is computationally complex, requiring the use of large memory resources due to its 2D Fast Fourier Transform operations. Thus, it is not suitable for deployment in consumer level digital cameras due to their limited cost and size requirements. An alternative to the Wiener filter would be to use a bilateral filter. The basic idea of the bilateral filter is to replace a pixel value in an image by a weighted mean of its neighbors, and the weights depend on both the spatial distance and the intensity distance [25,26]. The bilateral filter is a good choice for removing additive noise because it is stable and simple [27].

According to [28,29], some noise, for example speckle noise, cannot be modeled as additive noise. This kind of noise, modeled as multiplicative noise, is caused by the interaction between the ultrasound waves and the scatterers within the tissue [30]. As we stated previously in [31], in additive noise model, the difference between any two pixels from the same homogenous region is only related to the difference of the noise. Differently, in multiplicative noise, the difference between two pixels in the same homogenous region not only depends on the difference of the noise, but also depends on the intensity of the region. As a result, the image corrupted by multiplicative noise has different distributions in different homogenous regions. Traditional bilateral filters fail to handle this granular structure. In order to cope with multiplicative noise model, we use the modified bilateral filter proposed in [31], which can be described as follows:

(1) $$\bar{J}\left(X\right)=\frac{1}{C}\sum_{Y\in N\left(X\right)}e^{\frac{-{\left\|Y-X\right\|}^{2}}{2\sigma_{d}^{2}}}e^{\frac{-{\left\|J\left(Y\right)-J\left(X\right)\right\|}^{2}}{2\sigma_{r}^{2}{\left\|J\left(X\right)\right\|}^{2}}}J\left(Y\right)$$

where *J̄*(*X*) is the output value of a pixel, *J*(*Y*) is the input values of a pixel, *N*(*X*) is the set of spatial neighborhoods of pixel *X*, $\sigma_{d}^{2}$ and $\sigma_{r}^{2}$ are the parameters controlling the fall-off of weights in spatial and intensity domains, respectively, ‖ ‖ is Euclidean distance, C is used for the normalization and is defined as [27]:

(2) $$C=\sum_{Y\in N\left(X\right)}e^{\frac{-{\left\|Y-X\right\|}^{2}}{2\sigma_{d}^{2}}}e^{\frac{-{\left\|J\left(Y\right)-J\left(X\right)\right\|}^{2}}{2\sigma_{r}^{2}}}$$

### 3.2. Contrast Measure

A good contrast measurement should have enough discrimination ability over image sequence. As mentioned previously, various contrast measurements have been introduced and investigated in passive AF systems. The Contrast Measure based on Squared Laplacian (CMSL) yields good performance in terms of accuracy and unimodality [4,6,11]. It can be given as follows:

(3) $$L\left(x,y\right)=\frac{1}{J*K}\sum_{x=1}^{J}\sum_{y=1}^{K}G{\left(x,y\right)}^{2}$$

where *G*(*x*, *y*) is computed by:

(4) $$G\left(x,\text{ y}\right)=\sum_{i=x-1}^{x+1}\left|I\left(x,y\right)-I\left(i,y\right)\right|+\sum_{j=y-1}^{y+1}\left|I\left(x,y\right)-I\left(x,j\right)\right|$$

where *I*(*x*, *y*) is the intensity value of an image pixel at location (*x*, *y*), the parameters *J* and *K* are the height and width of focusing region in the image over which the contrast is evaluated.

**Figure 3.**
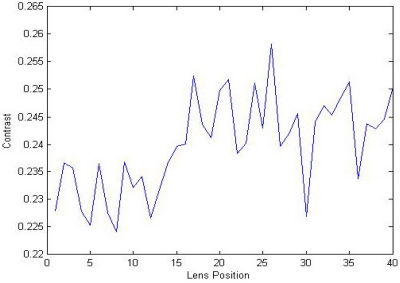
Influence of noise on the shape of a low contrast image.

However, under low light conditions, the higher frequency content in a focused image is just a little more than that in the corresponding defocused or blurred image. A focused image obtained from low light conditions possesses a small contrast value and it may be easily influenced by noise. Although we formerly used the modified bilateral filter for noise reduction, it is still necessary to cope with the influence of noise. As illustrated in Figure 3, due to the low contrast of image and noise influence, contrast measurement may generate a fluctuant curve with many local false peaks. In this case, it is difficult to distinguish a well-defined peak, and this hinders the following peak search procedure from performing its function in locating the best focused contrast peak.

As we know, the sharper the focus peak is, the easier it is to find the best focused lens position. Thus bringing in the local false peaks can decrease the noise contribution, and help locating the best focused contrast peak. According to [6], the second-order function gives better results than higher orders, because of the increase in noise effects. And it is better suited than the first-order difference, as the width of the top of the extremum of the focus function is smaller. Thus we wondered whether an order between first and second variance can be effective. In order to cope with noise and low light conditions, we introduce a contrast measure adaptive to noise influence (CMAN). Our contrast measure is defined based on Equation (3), which can be described as follows:

(5) $$F\left(x,\;y\right)=\frac{1}{J*K}\sum_{x=1}^{J}\sum_{y=1}^{K}G\left(x,y\right)\left(\sqrt[{n}]{G\left(x,y\right)+1}-1\right)$$

where n is computed by:

(6) $$n=\{\begin{array}{cc} 1 & m\leq T_{1}\\ 2 & T_{1}<m<T_{2}\\ 3 & m\geq T_{2} \end{array}$$

where the parameter m is the number of local maximums with value bigger than a predefined threshold T. When m is not more than the parameter T_1_, it means that the noise has little influence on image. We can observe a well defined peak standing for the best focused image. CMSL can work well on this image. In this case, the parameter n is equal to 1, and Equation (5) is equal to Equation (3). If m is more than the parameter T_1_ and less than the parameter T_2_, the noise adds a little bias to the contrast measure. At this time, the shape of the curve becomes a little fluctuant due to the noise. It becomes difficult to locate the focus peak due to the absence of a well defined peak. Decreasing the order of contrast measures can help alleviating the noise effects, and bring in local false peaks. As the number of local maxima with big values increases to a certain extent T_2_, the shape of the curve becomes more fluctuant, and it becomes more difficult to locate the best focused image. In this case, a further decrease of the order of contrast measures is necessary.

CMAN is theoretically self-consistent. For a continuous function such as Equation (5), its left derivative has the same value to its right derivative from the point *G*(*x*, *y*) = 0 to *G*(*x*, *y*) → +∞. And from Equation (4), we can confirm that *G*(*x*, *y*) ≥ 0. According to the definition of derivability [32], it can thus be derived that Equation (5) has the function derivability on each point, for example the point *x*_0_. Then it is also differential on the point *x*_0_ [32]. We can then obtain that [32]:

(7) $$\mathrm{dy}=f^{\prime }\left(x_{0}\right)\cdot \Delta x$$

As stated in [32], if Δ*x* is quite small, and under the assumption that ƒ'(*x*_0_) ≠ 0, it can be derived that:

(8) dy≈Δy

By using Equation (7) in Equation (8), it can be derived:

(9) $$f^{\prime }\left(x_{0}\right)\cdot \Delta x\approx f\left(x+x_{0}\right)-f\left(x_{0}\right)$$

and it can be also described as:

(10) $$f\left(x+x_{0}\right)\approx f\left(x_{0}\right)+f^{\prime }\left(x_{0}\right)\cdot \left(x-x_{0}\right)$$

If a pixel *x* in an image is from the same homogenous region with its adjacent pixels *y_n_* (n = 1, 2, …, 8), then we have *I*(*x*) = *I*(*y_n_*). From Equation (4), it can be derived that *G*(*x*, *y*) = 0. Thus we set *x*_0_ = 0 in Equation (10), and it can be derived that:

(11) $$f\left(x\right)\approx f\left(0\right)+f^{\prime }\left(0\right)\cdot x$$

By using Equation (11) in our proposed contrast measure Equation (5), it can be derived that:

(12) $$\begin{array}{ll} F\left(x,y\right) & =\frac{1}{J*K}\sum_{x=1}^{J}\sum_{y=1}^{K}G\left(x,y\right)\left(\sqrt[{n}]{G\left(x,y\right)+1}-1\right)\\ & \approx \frac{1}{J*K}\sum_{x=1}^{J}\sum_{y=1}^{K}G\left(x,y\right)*\left(\left(\sqrt[{n}]{0+1}-1\right)+\left(\frac{1}{n}{\left(0+1\right)}^{\frac{1}{n}-1}\right)G\left(x,y\right)\right)\\ & =\frac{1}{J*K*n}\sum_{x=1}^{J}\sum_{y=1}^{K}G{\left(x,y\right)}^{2}=\frac{1}{n}L\left(x,y\right) \end{array}$$

By using Equation (6) in Equation (12), and under the assumption that pixels in image are from the same homogenous region with its adjacent pixels, it can be derived that:

(13) $$F\left(x,y\right)\approx \{\begin{array}{cc} L\left(x,y\right) & m\leq T_{1}\\ \frac{1}{2}L\left(x,y\right) & T_{1}<m<T_{2}\\ \frac{1}{3}L\left(x,y\right) & m\geq T_{2} \end{array}$$

Equation (13) means that the value of CMAN decreases when the noise contributions increase. When an image is little influenced by noise, CMAN has almost the same value as CMSL. As stated previously, when noise adds a little bias to the image, the shape of the curve becomes a little fluctuant. In this case, the value of CMAN decreases to almost half the value of CMSL. This means a decrease of the value of local false peaks, and therefore the curve shape becomes flat in the homogenous region. When noise adds more bias to the image, CMAN has even less value than CMSL. As a result, CMAN brings in the peak of local maximum caused by the noise influence and a well-defined focus peak can thus be produced.

### 3.3. Peak Search

While the focusing accuracy is dependent on the contrast measure used, the reliability and speed of an AF algorithm depend on the peak search algorithm. Various peak search algorithms were presented in the past, such as global search, Fibonacci search [11], coarse-to-fine search [33], and rule-based search [5]. For a low contrast image, the contrast difference of the images obtained in different lens positions is quite small. It is difficult to decide when it is suitable to search the focus range using a big step size, thus coarse-to-fine search and rule-based search cannot work well in low light conditions. Global search scans the entire focus range with the smallest motor step in a forward manner. The main advantage of this algorithm is that there is no possibility of falsely obtaining a local peak since all focus positions are examined. However, each evaluation of the criterion function is a relatively expensive operation; it needs to minimize the number of evaluations required. For this reason, global search is not a feasible strategy. Fibonacci search uses the Fibonacci numbers to scan lens positions, and can achieve good convergence speed. However, it reverses the search direction too many times and thus possesses high power consumption [5].

In order to make the peak search robust to low light conditions, tradeoffs in terms of focusing reliability, convergence speed, and power consumption should be made. We adopt the AF peak search algorithm proposed by Li in [1], which takes account of the limited resolution of human visual systems. As stated in [1], the focus is acceptable when it is 12 μm (corresponding to four times the smallest moving step) off the peak. Thus we scan the entire focus range in a forward direction using four motor steps at each movement. Departing from Li who used stop criteria to expedite the convergence speed, the peak search procedure will not terminate until the last lens position to ensure the focusing reliability under low light conditions. This peak search has enough convergence speed because it is not an exhaustive search. In addition, because the search does not always start from the home position; therefore it could avoid blur experience and saves energy [1].

### 3.4. Procedure of the Proposed AF Algorithm

**Figure 4.**
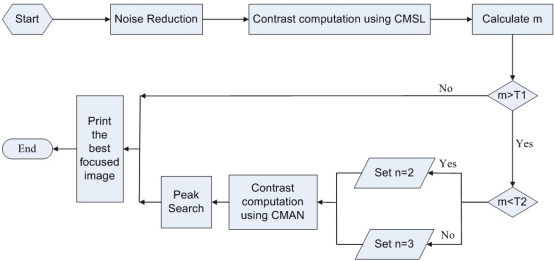
Proposed passive AF algorithm using CMAN.

As illustrated in Figure 4, after the noise reduction preprocessing, contrasts of image sequence are computed using CMSL. We can then calculate the number of local maxima with big contrast values. As a result, the parameter m can be obtained. If m is less than the parameter T_1_, it can observe a well defined peak with a maximum contrast value standing for the best focused image. Otherwise, we compare m to the parameter T_2_ to obtain the value of n, and calculate the image contrast using CMAN according to the parameter n. After that, a peak search procedure is performed to locating the best focused image. In this case, CMAN can alleviate the noise influence, and at the same time produce a well-defined focus peak. Although the contrast computation is a little more complex than CMSL, the computation time is acceptable and will not cause any lag in our passive AF system. In addition, the dedicated hardware architecture for real-time auto-focusing, such as field programmable gate array (FPGA)-based AF system [34], can be used to shorten the computation time, which processes the incoming pixels simultaneously with their neighboring pixels based on its parallelized window processing architecture. The extra computation in CMAN aims to distinguish the best focused peak from noise influence which is critical to ensure the focusing accuracy and reliability. Thus it is a suitable measurement for gauge the effectiveness of our passive AF algorithm in improving the performance under low light conditions.

## 4. Experiments and Results

**Figure 5.**
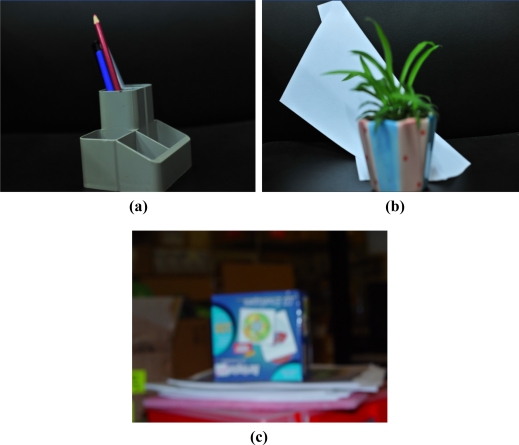
Image used in the experiments **(a)** Object A. **(b)** Object B. **(c)** Object C.

In the experiments, the objects A, B, and C (Figure 5) were autofocused in different lens positions using our proposed passive AF algorithm, from defocusing to focusing and from focusing to defocusing. Three different image sequences can be obtained, which were used to investigate the performance of CMAN. The three image sequences are representative for a certain class of image types, including low contrast image with little noise influence (object A), low contrast image with adequate noise (object B) and low contrast image corrupted by much noise (object C). The noise can be either additive noise or multiplicative noise. CMAN and CMSL were tested in the experiments, where T is set to be half value of the focused peak in CMSL, T_1_ is set to be 1 indicating just one well defined peak, and T_2_ is set to be 9 standing for the presence of adequate noise. In order to evaluate the performance of CMAN and CMSL, noise reduction preprocessing and peak search procedure were implemented in all of the tests. From the previous discussion, it can be expected that CMAN and CMSL would response differently towards these image sequences.

**Figure 6.**
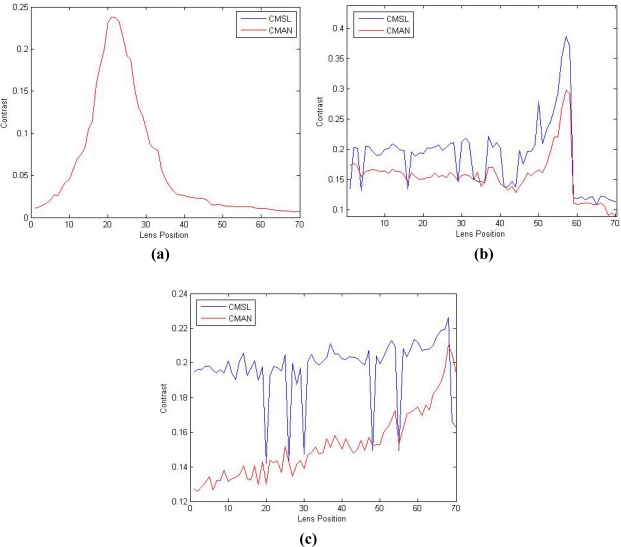
The shape of a low contrast image obtained by CMSL and CMAN **(a)** under little noise conditions; **(b)** under adequate noise conditions; **(c)** under much noise conditions.

In the first experiment, the resulting contrast value is plotted against 70 different lens positions in a consumer level digital camera, from near to far. As illustrated in Figure 6(a), CMSL and CMAN have the same contrast value in all the 70 lens positions when the image is little influenced by noise. And it can witness a well defined focus peak in the 21st lens position.

In the second experiment, the low contrast image with adequate noise influence acquired at 70 different lens positions from near to far is used for this experiment. The results are shown in Figure 6(b), where the 57th lens position is the one with the best contrast. As we can see, when noise has a little influence on the image, the curve generated by CMSL is more fluctuant than CMAN and we can observe a local false peak with a big value in the 50th lens position of the curve produced by CMSL. In this case, it is difficult to distinguish the focus peak from this local false peak. Differently, CMAN can decrease the value of local false peak and works well in this image. As illustrated in Figure 6(b), although there are still a number of local false peaks, their value is relatively small and they can be distinguished from the focused peak. In other words, CMAN brings in the peak of local maximum caused by the noise influence, and produces a well-defined focus peak.

In the last experiment, the low contrast image is corrupted by much noise. We also capture the image sequence at 70 different lens positions from near to far. The 68th lens position is the one with the best contrast. The results shown in Figure 6(c) clearly indicate that CMAN has more robust performance than CMSL. While CMSL fails, CMAN is still able to determine correctly the focus position.

## 5. Conclusions

This paper has described a robust passive AF algorithm using a new contrast measurement. The proposed algorithm is robust to both additive noise and multiplicative noise, and can provide a noticeable improvement in discrimination performance under low light conditions. The experimental results from an actual digital camera deployment validate the theory developed to distinguish the focus peak from local false peaks.
